# Acral Speckled Lentiginous Nevus

**DOI:** 10.7759/cureus.98813

**Published:** 2025-12-09

**Authors:** Natalia Maverakis Ramirez, Zachary J Jaeger, Hadas Skupsky, Anne Marie McNeill, Antoanella Calame

**Affiliations:** 1 School of Medicine, San Juan Bautista School of Medicine, Caguas, USA; 2 Dermatology, University of California San Diego, San Diego, USA; 3 Dermatology/Dermatopathology, Compass Dermatopathology, San Diego, USA; 4 Dermatology, Newport Beach Dermatology and Plastic Surgery, Newport Beach, USA; 5 Dermatology, Scripps Memorial Hospital, San Diego, USA

**Keywords:** acral nevus, compound nevus, congenital nevus, melanocytic nevus, melanoma, nevi spili, nevus spilus, special site nevus, speckled lentiginous nevus

## Abstract

Speckled lentiginous nevus (SLN), also referred to as nevus spilus, is a common benign melanocytic neoplasm typically occurring as a small, café-au-lait-colored “speckled” patch studded with numerous darkly pigmented macules or papules. Herein, we present a unique case of SLN arising on the sole of the right foot in a young woman, which was reported to be gradually enlarging since a pregnancy two years prior. The lesion was removed by shave biopsy, and histopathology confirmed the diagnosis and ruled out atypical features. While SLN is quite common, the occurrence on acral skin is extremely rare. SLN is considered a mosaic RASopathy due to its embryological development through a postzygotic activating HRAS genetic variant. Although small lesions usually remain isolated, underlying SLN syndrome should be considered in extensive cases with associated neurologic or musculoskeletal abnormalities. Secondary melanocytic neoplasms commonly arise within SLN, but overall, small lesions carry a low risk of malignant transformation, and prophylactic surgical treatment is not necessary. We briefly discuss SLN occurring in special sites and report this novel case with a review of the literature on SLN.

## Introduction

Speckled lentiginous nevus (SLN), also referred to as nevus spilus, is a common benign melanocytic neoplasm typically occurring as a small, isolated patch studded with numerous darkly pigmented macules. Macular SLN tends to have an evenly distributed checkerboard pattern of pigmented macules throughout the nevus [1]. The reported prevalence is around 1-3%, similar to that of congenital melanocytic nevi [1,2]. Here, we present a unique case of SLN arising on an acral surface and briefly discuss SLN occurring in special sites, as nevi on special sites have historically posed diagnostic challenges.

## Case presentation

**Figure 1:**
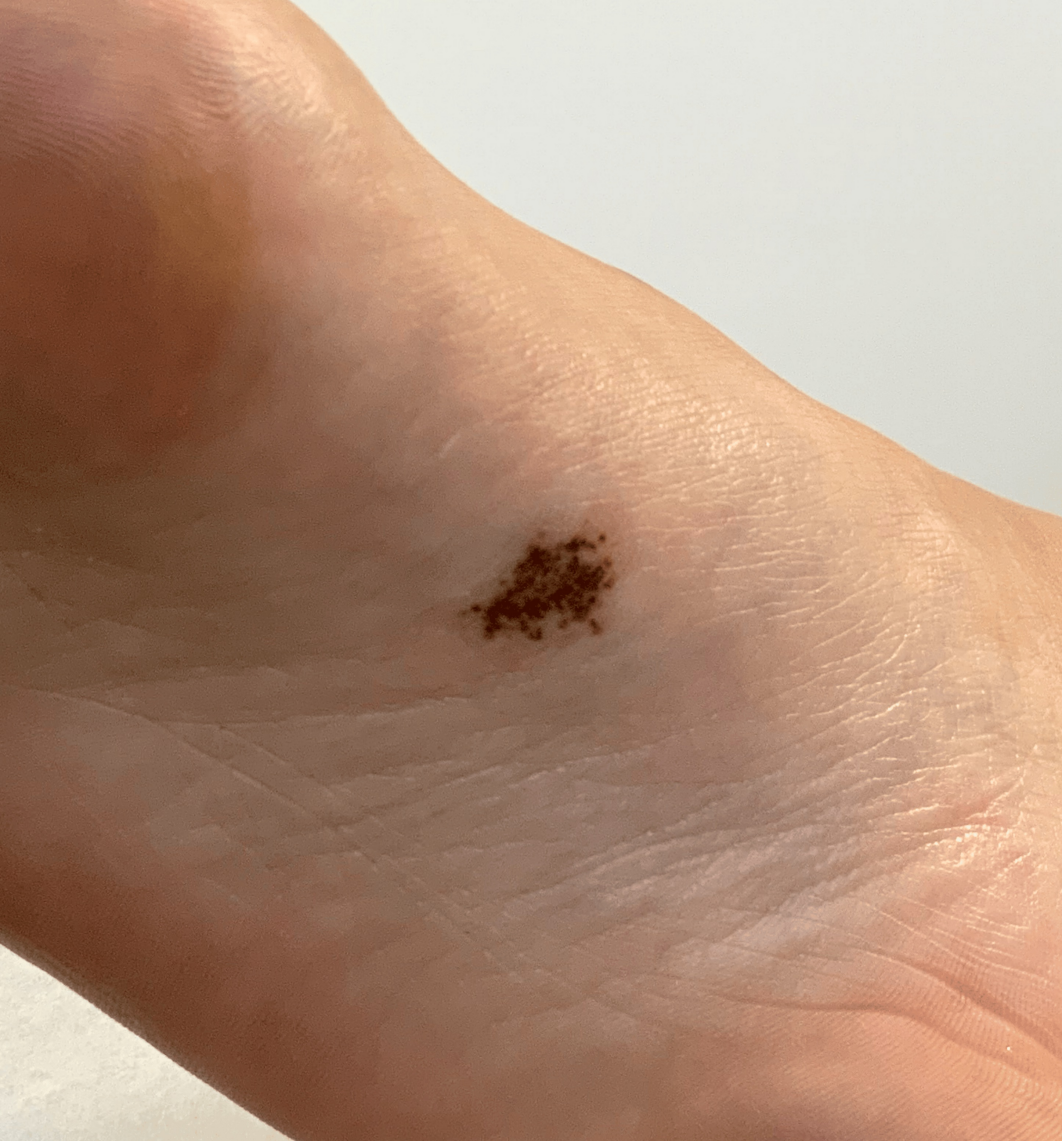
Acral speckled lentiginous nevus A tan patch on the sole of the right foot studded with numerous coalescing dark brown macules.

**Figure 2:**
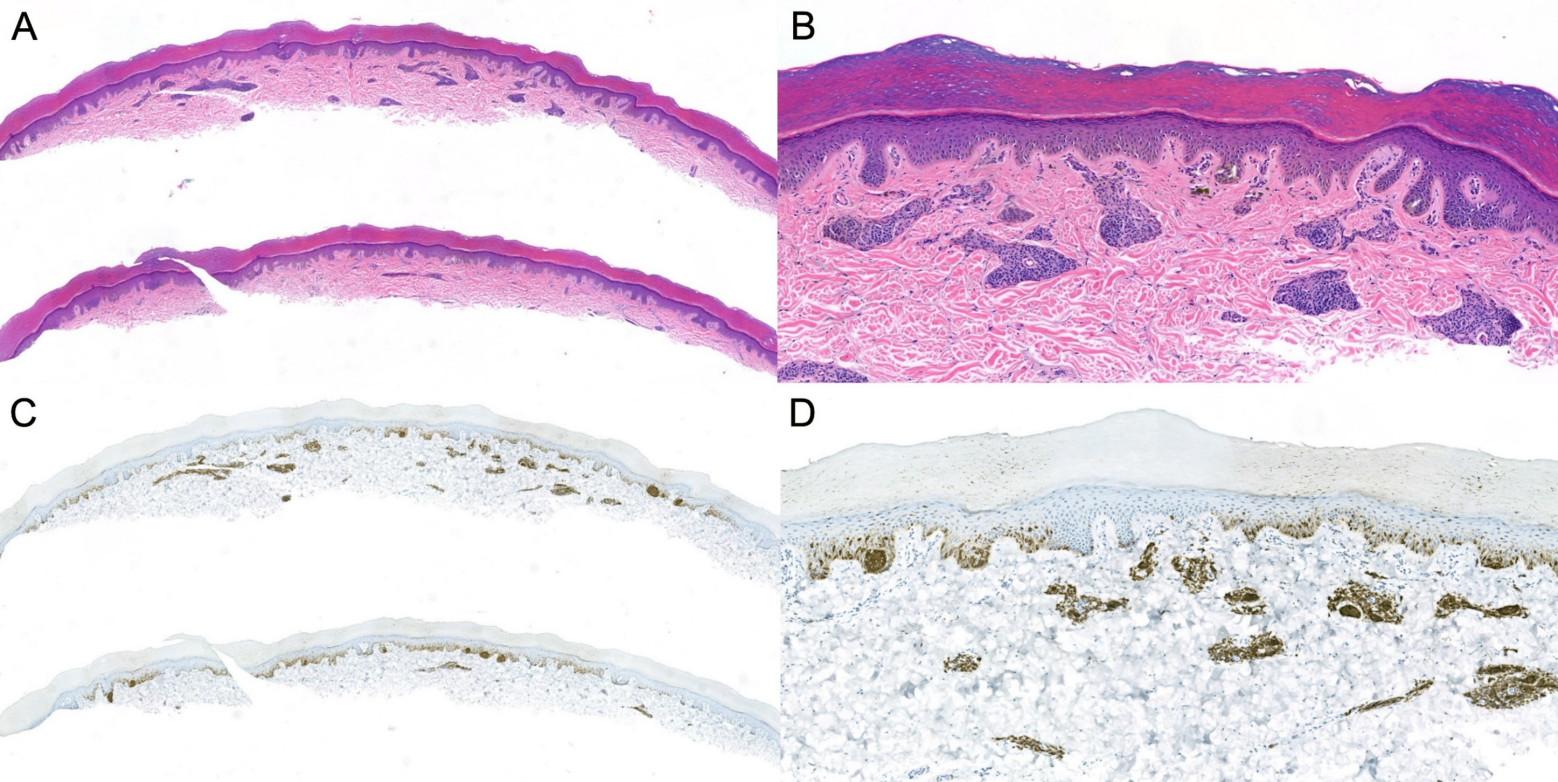
Acral speckled lentiginous nevus on routine histopathology Acral speckled lentiginous nevus on routine histopathology stained with hematoxylin and eosin (H&E) at low magnification (A, 50x) and higher power (B, 200x): a compound melanocytic proliferation with compact nests of bland nevus cells in clusters throughout the dermis and at the tips of rete ridges along with lentiginous junctional growth, without features of cytologic atypia or architectural disorder. Immunohistochemistry targeting melanoma antigen recognized by T cells 1 (MART-1) demonstrates the distribution of melanocytes in the nevus and highlights regular columns of melanin pseudoparakeratosis overlying junctional melanocyte nests (C, 50x; D, 200x).

A 28-year-old woman with a history of two basal cell carcinomas presented to the dermatology clinic for an asymptomatic birthmark on the sole of the right foot, gradually enlarging since a pregnancy two years ago (Figure 1). A shave biopsy was performed to sample a portion of the lesion. On hematoxylin and eosin (H&E) staining, the lesion demonstrated a benign compound histomorphology, with congenital dermal features and a lentiginous junctional component containing scattered nests and minimal site-related cytologic atypia (Figures 2A-2D). Regular columns of melanin peppered the stratum corneum. Preferentially expressed antigen in melanoma (PRAME) immunohistochemistry was negative. Therefore, the diagnosis of benign acral nevus spilus was rendered.

## Discussion

Our patient’s case of acral SLN adds an interesting special-site SLN to the literature. To date, there is only one other reported case of an acral SLN on the heel of a child with a benign dermoscopic lattice pattern of pigment in the furrows [3]. SLN occurs predominantly on the trunk and lower extremities, with few cases occurring on special sites. A handful of rare cases of intraoral SLN have been reported [4]. The clinical, histopathological, and dermoscopic features of SLN are well established for non-acral skin, and the expected features on acral skin can be inferred from the established patterns of acral nevi. However, the extreme rarity of acral SLN in the literature underscores the novelty and clinical significance of this newly reported case.

The dermoscopic features of SLN in acral locations have not been directly described in the literature, as no published case reports or series document such cases. However, the dermoscopic patterns of acral melanocytic nevi are well established. The most common benign pattern is the parallel furrow pattern, in which pigmentation follows the furrows of the skin markings. Other benign patterns include the lattice-like, fibrillar, globular, and crista-dotted patterns. The parallel ridge pattern, in which pigmentation is located on the ridges, is highly specific for early acral lentiginous melanoma and is rarely seen in benign nevi. In the context of SLN, the expected dermoscopic appearance on acral skin would be a benign acral nevus background (most likely parallel furrow or lattice-like) with superimposed globules, dots, or papules corresponding to the speckles. This composite pattern would distinguish acral SLN from solitary acral nevi, which typically display a uniform dermoscopic architecture. The presence of a parallel ridge pattern, asymmetry, or multicomponent features should prompt concern for melanoma. 

The leading hypothesis for the origin of SLN involves a mosaic pattern of embryonic development with genetic variants, notably HRAS, responsible for the patchy or speckled appearance and tendency to respect the cutaneous lines of Blaschko [5]. Both conventional and phacomatosis pigmentokeratotica-associated SLN have been observed to exhibit a postzygotic activating HRAS genetic variant that over-activates the RAS-MAPK pathway [6]. However, secondary neoplasms within these lesions do not all show the same molecular mechanism of pathogenesis [7]. This finding suggests that a second genetic aberrancy within the affected cutaneous segment may be necessary to engender neoplastic behavior in the individual melanocytes [8]. Nevus spilus-type congenital melanocytic nevi have also been found to harbor an activating NRAS variant, though this entity is a genotypically and phenotypically distinct class of mosaic RASopathies [9,10]. Further genomic analyses are needed to elucidate the molecular pathogenesis of these melanocytic lesions. 

Secondary neoplasia within SLN is well documented. Numerous instances of benign neoplasms have been reported to arise in pre-existing SLN, including Spitz nevi, blue nevi, and lentigines [2]. However, the precise epidemiology of melanocytic dysplasia within SLN remains controversial. The incidence of melanoma arising in SLN has been described in case reports but is rare and considered to be proportional to the lesion size [2,11]. A few reports have documented cases of multiple melanomas occurring within the same SLN [11]. For small-to-medium-sized congenital melanocytic nevi, the risk of developing melanoma is estimated at less than 1% [12]. However, the risk for larger lesions appears to be non-negligible and thus warrants clinical observation for changes over time [13]. 

Due to the association of SLN with neurocutaneous syndromes, it is important to screen patients for related extracutaneous features. Predominantly macular SLN is associated with phacomatosis pigmentovascularis type III alongside a port wine stain with or without nevus anemicus, as well as the similar type IV (phacomatosis spilorosea) with the addition of dermal melanocytosis. Predominantly papular SLN comprises a necessary component for the diagnosis of phacomatosis pigmentokeratotica (also coined as phacomatosis spilosebacea to accurately describe its mosaic components) and SLN syndrome (also referred to as papular nevus spilus syndrome, occurring in the setting of ipsilateral neurologic or musculoskeletal deficits) [14,15]. A systematic review on melanocytic neoplasms in patients with neurofibromatosis type 1 found that nearly half of those with nevi also exhibited SLN [16]. 

Due to the benign nature of these nevi, preemptive excision is not recommended. However, in the event of clinically apparent changes or markedly atypical microscopic features, surgical treatment may be advisable but can be difficult to achieve, especially for larger lesions and those studded with numerous additional neoplasms. Studies have described variable success with cosmetic destruction by melanin-targeting lasers [17]. Given the relation to mosaic RASopathies involving hyperactivation of the RAS-MAPK-MEK pathway, MEK inhibitors have shown some initial promise in case reports of congenital melanocytic lesions with activating genetic variants in NRAS and mosaic BRAF fusions [18-20]. Severe or symptomatic cases may warrant further genomic analysis and potentially a trial of RAS-MAPK-MEK pathway inhibition. 

## Conclusions

In summary, we present an extremely rare case of SLN arising on the plantar foot. SLN is considered a mosaic RASopathy due to its embryological development, and underlying SLN syndrome should be considered in extensive cases. Although secondary melanocytic neoplasms commonly arise within SLN, small lesions carry a low risk of malignant transformation. However, SLN is benign and prophylactic surgical treatment is not necessary.

## References

[1] Vidaurri-de la Cruz H, Happle R (2006). Two distinct types of speckled lentiginous nevi characterized by macular versus papular speckles. Dermatology.

[2] Schaffer JV, Orlow SJ, Lazova R, Bolognia JL (2001). Speckled lentiginous nevus: within the spectrum of congenital melanocytic nevi. Arch Dermatol.

[3] Valdivielso-Ramos M, Mauleon C, E. Balbín E, de la Cueva P, Chavarría E, Hernanz JM (2009). Congenital plantar nevus spilus. Acta Pediatr Esp.

[4] Torres KG, Carle L, Royer M (2017). Nevus spilus (speckled lentiginous nevus) in the oral cavity: report of a case and review of the literature. Am J Dermatopathol.

[5] Groesser L, Herschberger E, Sagrera A (2013). Phacomatosis pigmentokeratotica is caused by a postzygotic HRAS mutation in a multipotent progenitor cell. J Invest Dermatol.

[6] Zhang J, Xu Q, Deng D (2023). Genetic and phenotypic diversities of nevus spilus phenotypes: case series and a proposed diagnostic algorithm. Clin Genet.

[7] Sarin KY, McNiff JM, Kwok S, Kim J, Khavari PA (2014). Activating HRAS mutation in nevus spilus. J Invest Dermatol.

[8] Jaeger ZJ, Maverakis Ramirez N, Osborne AD (2025). RASopathies. Part I: genetics and therapeutic considerations. J Am Acad Dermatol.

[9] Kinsler VA, Krengel S, Riviere JB (2014). Next-generation sequencing of nevus spilus-type congenital melanocytic nevus: exquisite genotype-phenotype correlation in mosaic RASopathies. J Invest Dermatol.

[10] Jaeger ZJ, Maverakis Ramirez NK, Osborne AD, Staser KW, King KA, Bayliss SJ, Mann C (2025). RASopathies. Part II: cutaneous and extracutaneous manifestations. J Am Acad Dermatol.

[11] Corradin MT, Zattra E, Fiorentino R, Alaibac M, Belloni-Fortina A (2010). Nevus spilus and melanoma: case report and review of the literature. J Cutan Med Surg.

[12] Krengel S, Hauschild A, Schäfer T (2006). Melanoma risk in congenital melanocytic naevi: a systematic review. Br J Dermatol.

[13] Lovett A, Maari C, Decarie JC (2009). Large congenital melanocytic nevi and neurocutaneous melanocytosis: one pediatric center's experience. J Am Acad Dermatol.

[14] Happle R (2002). Speckled lentiginous nevus syndrome: delineation of a new distinct neurocutaneous phenotype. Eur J Dermatol.

[15] Torchia D, Happle R (2023). Phacomatosis spilosebacea: a new name for a distinctive binary genodermatosis. J Am Acad Dermatol.

[16] Meyer SN, Simmons E, Studer AC, Rauen KA, Kiuru M (2023). Melanocytic neoplasms in neurofibromatosis type 1: a systematic review. Melanoma Res.

[17] Han HT, Park JJ, Lee JS, Lee SH (2024). Nevus spilus, partial unilateral lentiginosis, and linear and whorled nevoid hypermelanosis: a comparison of clinical features, course, and treatment response. Acta Derm Venereol.

[18] Krengel S, Widmer DS, Kerl K, Levesque MP, Schiestl C, Weibel L (2016). Naevus spilus-type congenital melanocytic naevus associated with a novel NRAS codon 61 mutation. Br J Dermatol.

[19] Martin SB, Polubothu S, Bruzos AL (2024). Mosaic BRAF fusions are a recurrent cause of congenital melanocytic nevi targetable by MAPK pathway inhibition. J Invest Dermatol.

[20] Berna R, Hasbun T, Adams D, Moon AT, Treat JR (2024). Significant improvement of a nevus spilus-type congenital melanocytic nevus with oral selumetinib. Pediatr Dermatol.

